# Genetic Insights and Neonatal Outcomes in Preeclampsia and Eclampsia: A Detailed Analysis of the RS5707 Genotype

**DOI:** 10.3390/diagnostics14131366

**Published:** 2024-06-27

**Authors:** Flavius George Socol, Elena Silvia Bernad, Marius Craina, Simona-Alina Abu-Awwad, Brenda-Cristiana Bernad, Ioana Denisa Socol, Simona Sorina Farcas, Ahmed Abu-Awwad, Nicoleta Ioana Andreescu

**Affiliations:** 1 Doctoral School, “Victor Babes” University of Medicine and Pharmacy, Eftimie Murgu Sq. No. 2, 300041 Timisoara, Romania; george.socol@umft.ro (F.G.S.); bernad.brenda@umft.ro (B.-C.B.); ioana.socol@umft.ro (I.D.S.); 2 Ist Clinic of Obstetrics and Gynecology, “Pius Brinzeu” County Clinical Emergency Hospital, 300723 Timisoara, Romania; bernad.elena@umft.ro (E.S.B.); craina.marius@umft.ro (M.C.); 3 Department of Obstetrics and Gynecology, Faculty of Medicine, “Victor Babes” University of Medicine and Pharmacy, 300041 Timisoara, Romania; 4 Center for Laparoscopy, Laparoscopic Surgery and In Vitro Fertilization, “Victor Babes” University of Medicine and Pharmacy, 300041 Timisoara, Romania; 5 Center for Neuropsychology and Behavioral Medicine, “Victor Babes” University of Medicine and Pharmacy, 300041 Timisoara, Romania; 6 Department of Microscopic Morphology—Genetics, Center of Genomic Medicine, “Victor Babes” University of Medicine and Pharmacy, Eftimie Murgu Sq. No. 2, 300041 Timisoara, Romania; farcas.simona@umft.ro (S.S.F.); andreescu.nicoleta@umft.ro (N.I.A.); 7 Department XV—Discipline of Orthopedics—Traumatology, “Victor Babes” University of Medicine and Pharmacy, 300041 Timisoara, Romania; ahm.abuawwad@umft.ro; 8 Research Center University Professor Doctor Teodor Sora, “Victor Babes” University of Medicine and Pharmacy, 300041 Timisoara, Romania

**Keywords:** preeclampsia, eclampsia, rs5707 polymorphism, genetic predisposition, maternal health, neonatal outcomes, pregnancy complication

## Abstract

Background: Preeclampsia (PE) and eclampsia (E) are severe pregnancy complications with significant maternal and neonatal health impacts. This study explores the association of the rs5707 polymorphism in the renin-angiotensin system (RAS) with PE/E and related neonatal outcomes. Materials and Methods: We conducted a cross-sectional study involving 400 mother–newborn dyads at the “Pius Brinzeu” Emergency Clinical Hospital Timisoara. Participants were divided into a control group (254 normotensive women) and a PE/E group (146 women with PE/E). Genotyping for the rs5707 polymorphism was performed using real-time PCR, and statistical analyses assessed associations with maternal body mass index (BMI) and neonatal outcomes. Results: The AA genotype of rs5707 was significantly associated with a reduced risk of PE/E and more favorable neonatal outcomes, including higher Apgar scores, greater birth weights, and longer gestational ages. Conversely, the AC genotype correlated with increased maternal BMI and adverse neonatal outcomes. Odds ratios highlighted the protective effect of the AA genotype against PE/E and the increased risk associated with the AC genotype. Conclusions: This study revealed the critical role of the rs5707 polymorphism in PE/E development and neonatal health. Genetic screening for rs5707 could enhance early identification and personalized intervention strategies, improving outcomes for both mothers and neonates. Further research is needed to validate these findings across diverse populations and to uncover the underlying mechanisms.

## 1. Introduction

Preeclampsia (PE), an obstetrical complication characterized by the development of hypertension and organ dysfunction [1], often indicated by the presence of proteinuria, accounts for 2 to 8% of pregnancy-related complications, contributing to over 50,000 maternal deaths and more than 500,000 fetal deaths worldwide. In Western countries, studies show that Spain records the lowest incidence of PE, with rates ranging from 0.7 to 1.6%, whereas Finland experiences the highest, between 3.6 and 8.1% [2].

Eclampsia, a severe manifestation of preeclampsia, poses significant risks to both the mother and the fetus. Rapid recognition and immediate treatment of this condition are essential [3]. Eclampsia typically presents with seizures or convulsions triggered by PE and is a major health concern [4]. Without appropriate treatment, women with this condition can face severe complications, such as liver rupture, stroke, pulmonary edema, or renal failure [5,6]. Similarly, newborns can suffer from low birth weight, neonatal asphyxia, stillbirth, and even death [7,8].

The underlying mechanisms of PE/E remain incompletely understood, though the prevailing theory suggests that it could result from inadequate uteroplacental perfusion, caused by abnormal invasion of spiral arterioles [9]. The renin-angiotensin system (RAS) is one important component of this complete condition. As a sophisticated hormonal cascade, it plays an essential role in regulating blood volume, maintaining electrolyte balance, and controlling systemic vascular resistance [10]. However, RAS involvement is only one aspect of the multifaceted pathophysiology of PE.

Optimal uteroplacental circulation is vital for a successful pregnancy. It results from the systematic development and transformation of the entire uterine vascular system, including the formation of a vital fetal organ: the placenta. This transformation process is characterized by several cellular activities, such as cell proliferation and enlargement, reconfiguration of existing structures, and modifications in the extracellular matrix [11]. The behavior of the RAS in PE diverges from its normal function during a normal pregnancy. While typical pregnancies see a rise in circulating RAS components, women with PE exhibit lower levels of these components compared to those observed in women with normotensive pregnancies. This suggests that the RAS plays a significant role in the development of PE/E [12].

Angiotensin II, an octapeptide, is generated from angiotensinogen via consecutive enzymatic reactions involving renin and the angiotensin-converting enzyme (ACE). It serves as the primary active agent of the RAS. Angiotensin II not only elevates blood pressure but also prompts the renal tubules to conserve sodium and water and triggers the secretion of aldosterone from the adrenal glands. In addition to its powerful vasoconstrictive effects, angiotensin II also promotes cell growth, inflammation, and fibrosis [13]. It is also known that women with PE/E have lower levels of renin and angiotensin II but exhibit an increased sensitivity to angiotensin II, which enhances vasoconstrictive effects and further increases blood pressure [14].

Some variations in plasma renin levels have been linked to polymorphisms in the REN gene [15]. Previous studies have identified specific single-nucleotide polymorphisms (SNPs) associated with an increased risk of hypertension and PE/E [16,17,18,19,20]. This study investigates the association between rs5707 from the mother and the newborn and PE/E.

rs5707 is a specific single-nucleotide polymorphism (SNP) located within a gene of interest. SNPs are variations at a single position in a DNA sequence among individuals [21]. The presence of rs5707 indicates a variation that may be associated with particular traits or susceptibilities to certain diseases. Identifying and studying such SNPs can help in understanding genetic predispositions and the underlying mechanisms of various conditions, including preeclampsia [22].

Genetic analysis is complex, considering that the implications of both maternal and fetal genes must be evaluated simultaneously. Research has shown that the variation in preeclampsia (PE) can be attributed to multiple factors: 35% to maternal genetics, 20% to fetal genetics, 13% to paternal genetics, and 32% to environmental factors [23].

In this study, we adopted a cross-sectional approach to simultaneously assess the correlation between maternal and fetal genetic variants and the development of PE/E, while also considering the interaction with environmental factors. The primary goal was to demonstrate the association between the presence of the rs5707 genetic variant in the mother and the risk of developing PE/E.

## 2. Materials and Methods

### 2.1. Study Design and Participants

This study adopted a cross-sectional design, wherein data and biological samples were collected from mother–newborn dyads upon hospital admission for delivery and, respectively, within the first days of life for newborns. Pregnant women were recruited from the Clinical Departments of Obstetrics and Gynecology of the “Pius Brinzeu” Emergency Clinical Hospital Timisoara, between 2018 and 2023. A total of 400 pregnant women were included in the study, divided into 2 groups: Group 1 is named the “Control Group” and consisted of 254 pregnant women without documented associated pathologies, with blood pressure within normal parameters. Group 2 comprised 146 women diagnosed with preeclampsia or eclampsia.

Hypertensive disorders during pregnancy, impacting 10% of all pregnancies, are characterized by the International Society for the Study of Hypertension in Pregnancy (ISSHP) as the emergence of hypertension (systolic blood pressure ≥ 140 mmHg or diastolic blood pressure ≥ 90 mmHg) post 20 weeks of gestation. This broad categorization covers chronic hypertension, gestational hypertension, and preeclampsia, whether it is de novo or superimposed on existing chronic hypertension [24]. Eclampsia is characterized by the new onset of generalized tonic–clonic seizures in a patient with preeclampsia. These eclamptic seizures can manifest antepartum, from 20 weeks of gestation onwards, as well as during labor (intrapartum) and after delivery (postpartum). Although seizures occurring before 20 weeks of gestation are uncommon, they have been observed in cases of gestational trophoblastic disease [3].

To optimize the organization and clarity of the study, we established a set of criteria for including and excluding participants from this study (Table 1).

### 2.2. Data Collection

The route of delivery information was collected both through patient interviews and cross-verified with patient files to ensure accuracy. Regarding the stress level during pregnancy, it was quantified using the Perceived Stress Scale (PSS), a widely recognized tool for measuring the perception of stress. This scale was administered to all participants to obtain a standardized measure of stress levels during pregnancy.

Demographic data for the participants were gathered using a standardized questionnaire and personal interviews conducted by qualified medical personnel. The questionnaire covered maternal information, including age, educational level, marital status, and occupation, as well as obstetric data, such as gestational age at delivery, number of prior births, blood pressure, pre-pregnancy body mass index (BMI), classified according to standard BMI categories [25]: underweight (BMI < 18.5 kg/m^2^), normal weight (BMI 18.5–24.9 kg/m^2^), overweight (BMI 25–29.9 kg/m^2^), and obese (BMI ≥ 30 kg/m^2^), and family history of hypertension. Additionally, data on the weight of the newborn and stress levels during pregnancy were collected. This structured approach ensures the orderly presentation of the gathered data.

### 2.3. Genetic Screening and Polymorphism Analysis

Whole blood samples were collected from all participants, using EDTA tubes to preserve the samples. Genomic DNA was then extracted from these samples, employing the Genomic DNA Whole Blood Kit (Code 102 MagCore^®^) on the MagCore^®^ Plus II instrument from RBC Bioscience (New Taipei City, Taiwan). This procedure was performed following the protocol provided by the manufacturer.

After DNA isolation, the concentration and purity of the DNA samples were quantified using the BioTek Epoch microplate spectrophotometer (Agilent Technologies, Santa Clara, CA, USA). The DNA samples were diluted to a concentration of 10 ng/μL to ensure optimal conditions for subsequent analyses.

For the genotyping process, a reaction mixture was prepared, which included 10 μL of TaqMan Genotyping Master Mix, 1 μL of the TaqMan^®^ SNP Genotyping Assay (C__11942562_20), 8 μL of nuclease-free water, and 1 μL of the DNA template.

The genotyping experiments were conducted using the Applied Biosystems QuantStudio 7 Flex system, a state-of-the-art real-time PCR (Thermo Fisher Scientific, Carlsbad, CA, USA).

The allelic discrimination data were subsequently analyzed using the instrument’s software, which plotted the results to compare the signals from allele 1, marked with VIC dye, and allele 2, marked with FAM dye. This comparative analysis enabled the identification and differentiation of the alleles, ensuring reliable genotyping results.

### 2.4. Statistical Analysis

In our study, the statistical analysis was performed using MedCalc and GraphPad Prism 6, both of which are advanced statistical tools frequently used in biomedical research. These programs enabled a meticulous assessment of our data. We employed both the Z-test and the T-test in our statistical approach. The Z-test was utilized to evaluate differences in the proportions of categorical variables across two distinct groups, offering insights into variations in genotype frequencies. Before applying the *t*-test, we conducted a normality test using the Kolmogorov–Smirnov test, which is appropriate for our data groups. The *t*-test, on the other hand, was used to assess differences in the averages of continuous variables, helping us understand significant variations in physiological parameters associated with PE among various genotypes. Statistical significance was established with a *p*-value of less than 0.05, a standard threshold used in all our statistical tests to discern significant relationships and variances within our dataset. Additionally, we used SPSS software to enhance our data analysis capabilities, which supported complex statistical operations. We calculated odds ratios (ORs) with 95% confidence intervals (CIs) to determine the association between specific genotypes (GG, AG, and AA) and the risk of developing PE. These ORs helped quantify the probability of developing PE/E for each genotype relative to the reference group.

### 2.5. Ethical Consideration

In strict accordance with ethical guidelines, every participant in this study voluntarily provided informed consent before their inclusion. This informed consent process ensured that participants were fully aware of the study’s objectives and methods. Participants agreed to participate in the research, permitted the collection of their blood samples for scientific analysis, and authorized the use of their personal and health-related information for the study’s purposes. The thorough and transparent nature of this consent process was vital in respecting the autonomy and dignity of the participants, ensuring their rights and preferences were fully upheld.

Furthermore, the study implemented rigorous measures to safeguard the confidentiality and privacy of all participants. To protect their identities, all collected data were meticulously anonymized, removing any personal identifiers. This step was essential in demonstrating our unwavering commitment to upholding the highest ethical standards in research.

Additionally, the ethics committee of the “Pius Brînzeu” Clinical Emergency Hospital in Timișoara, Romania, granted ethical approval for this research. This approval, documented under number 72 on 28 June 2021, underscores our adherence to ethical research protocols and guidelines. The approval process involved a thorough review to ensure that all aspects of the study, including handling sensitive data and the informed consent process, met stringent ethical requirements. This comprehensive ethical oversight highlights our dedication to conducting research with integrity and respect for participant welfare.

## 3. Results

Various health and demographic variables between the control group (254 participants) and the PE/E group (146 participants) in the study on preeclampsia/eclampsia are compared in Table 2. Notably, the PE/E group had significantly higher maternal age and blood pressure (systolic and diastolic) than the control group, with *p*-values less than 0.0001, indicating strong statistical significance.

Educational levels, marital status, and occupation showed no significant differences between the two groups. However, there were significant differences in BMI before pregnancy and stress levels during pregnancy. The PE/E group had a higher proportion of participants with a pre-pregnancy BMI of ≥24 kg/m^2^ and reported higher levels of stress, with significant *p*-values for slight and high stress levels. These findings suggest that increased maternal age, higher blood pressure, elevated BMI, and higher stress levels are associated with PE/E.

The data presented in this study indicated that the AA and AC genotypes of the rs5707 polymorphism were associated with different risks of preeclampsia/eclampsia in both mothers and fetuses (Table 3). The AA genotype was associated with a reduced risk, while the AC genotype was associated with an increased risk. The CC genotype did not show a significant association with preeclampsia/eclampsia.

The distribution of each genotype (AA, AC, and CC) among participants with BMI < 24 kg/m^2^ and BMI ≥ 24 kg/m^2^ for both control and PE/E groups is presented in Table 4. The odds ratios (OR) and corresponding 95% confidence intervals (CI) indicate the likelihood of having a BMI ≥ 24 kg/m^2^ relative to the control group. Statistical significance was evaluated using *p*-values.

Table 5 illustrates the association of rs5707 genotypes (AA, AC, and CC) with neonatal outcomes, including Apgar score, birth weight, and gestational age at birth, comparing control and preeclampsia/eclampsia (PE/E) groups. The data present mean values along with standard deviations for each outcome measure, providing a comprehensive overview of how different genotypes impact these critical birth parameters. Statistical significance was assessed through *p*-values.

## 4. Discussion

This study investigated the association between rs5707 genotypes and various maternal and neonatal outcomes in both control and preeclampsia/eclampsia (PE/E) groups. The analysis highlighted significant genetic predispositions linked to the rs5707 genotype, influencing body mass index (BMI) before pregnancy and neonatal Apgar scores, birth weights, and gestational ages.

Our study highlighted differences in the distribution of rs5707 genotypes between the control group and the PE/E group, with a particular focus on pre-pregnancy BMI. Our findings indicated that the AA genotype, associated with a reduced risk of developing preeclampsia, was significantly more frequent in women in the control group with a BMI < 24 kg/m^2^. This aligns with existing literature suggesting the protective effect of the AA genotype against preeclampsia and other pregnancy-related complications [22]. In contrast, we observed that the AA genotype was considerably less common in women in the PE/E group with a BMI ≥ 24 kg/m^2^. This underscores the adverse role of overweight and obesity in exacerbating the risk of preeclampsia in women with genotypes that might otherwise offer protection. The literature also suggests that obesity is a major risk factor for preeclampsia, contributing to systemic inflammation [26] and endothelial dysfunction [27], which are amplified by genetic predisposition. The findings suggest a significant genetic predisposition linked to the rs5707 genotype, with the AA and AC genotypes showing particularly strong associations with increased BMI in the PE/E group. These results highlight the potential role of genetic factors in the development of obesity and overweight and its impact on pregnancy outcomes. Our results are consistent with previous studies that have demonstrated that the presence of the AA genotype may reduce the risk of developing preeclampsia through mechanisms involving blood pressure regulation and genetically modulated inflammatory responses [28].

The results may be inconclusive due to the small number of cases, which was insufficient to show a statistically significant difference. However, these findings suggest that a genetic profile could be performed to identify patients at risk for preeclampsia/eclampsia. No preventative or management strategies can be implemented as a result of this study. Still, the genetic evaluation highlights its importance in understanding the risk and could contribute to developing more effective strategies in the future.

The AC genotype was significantly associated with higher BMI in the PE/E group, suggesting it may predispose individuals to higher BMI and an increased risk of preeclampsia. Although less common, the CC genotype also showed a potential link to higher BMI, but the statistical significance was weaker.

These findings align with previous research indicating that genetic factors significantly contribute to the risk of preeclampsia and associated metabolic conditions. The substantial ORs for both AA and AC genotypes suggest that genetic screening for Rs5707 could be valuable in predicting and managing preeclampsia risk, especially in populations with high BMI [29].

The study further explored the impact of rs5707 genotypes on neonatal outcomes, focusing on Apgar scores, birth weights, and gestational ages at birth. The results demonstrated that neonates from the PE/E group generally had poorer outcomes compared to those from the control group, across all genotypes. Statistical significance was assessed through *p*-values. The results indicated significant differences in Apgar scores, birth weights, and gestational ages between the control and PE/E groups across the various genotypes. These findings underscore the potential influence of the rs5707 genotype on neonatal health and development, particularly in pregnancies complicated by PE/E.

Neonates with the AA genotype in the PE/E group had significantly lower Apgar scores, birth weights, and shorter gestational ages compared to the control group, with a highly significant *p*-value. These differences underscore the adverse effects of preeclampsia on neonatal health, even for those with a genotype typically associated with a lower risk of the condition.

Neonates with the maternal AC genotype in the PE/E group had poorer outcomes than those in the control group, with lower Apgar scores, reduced birth weights, and shorter gestational ages. These findings suggest that the AC genotype may worsen the negative impact of preeclampsia on neonatal outcomes.

Although less frequent, the CC genotype showed similar trends. Neonates in the PE/E group had lower Apgar scores, birth weights, and shorter gestational ages. Despite the weaker statistical significance for some measures, these trends indicate a detrimental effect of preeclampsia on neonates with the maternal CC genotype.

Neonates born to mothers with preeclampsia are at higher risk of adverse outcomes, with the severity influenced by genotype. The AA genotype showed the least adverse effects, while the AC genotype posed the most significant risks.

Our findings align with existing literature, which shows preeclampsia leads to poorer Apgar scores due to compromised placental function [30,31,32,33] and fetal hypoxia [34], reinforcing its adverse impact on neonatal health, even among lower-risk genotypes.

Neonates with the maternal AA genotype in the PE/E group had significantly lower birth weights, reflecting intrauterine growth restriction (IUGR) due to impaired placental perfusion. This aligns with existing literature documenting the link between preeclampsia and IUGR, leading to lower birth weights [35].

Gestational ages were shorter in the PE/E group, consistent with preeclampsia’s known association with preterm birth due to maternal or fetal distress, as supported by previous studies [36].

The associations found in this study suggest that genetic screening for rs5707 could help identify women at higher risk of preeclampsia, particularly those with a higher BMI. Early identification could enable targeted interventions, such as lifestyle changes, closer monitoring, and potentially earlier delivery to reduce risks to both the mother and fetus.

This study offers groundbreaking insights into preeclampsia (PE) and eclampsia (E), significantly enhancing our understanding of these conditions. A major highlight of this research was its examination of the rs5707 polymorphism within the renin-angiotensin system (RAS) and its impact on both maternal and neonatal health. Notably, we found that the AA genotype of rs5707 was associated with a lower risk of PE/E and better neonatal outcomes, while the AC genotype was linked to a higher maternal body mass index (BMI) and poorer neonatal outcomes. Additionally, the findings emphasize the potential of genetic screening to improve early detection and intervention for PE/E, promoting a personalized approach to treatment. By analyzing data from 400 mother–newborn pairs, this research provided valuable insights that could lead to more effective prevention and management strategies in clinical settings, ultimately benefiting the health of both mothers and their newborns.

## 5. Limitations and Future Research

Despite the significant findings, this study has several limitations that should be addressed in future research. First, the cross-sectional design limited the ability to establish causal relationships between rs5707 genotypes and the observed outcomes. Longitudinal studies would provide more robust data on how these genotypes influence the progression of preeclampsia and its effects on neonatal health over time.

Second, the study population was recruited from a single hospital, which may limit the generalizability of the findings. Future studies should include a more diverse population from multiple centers to validate these results across different demographic and geographic settings.

Third, while the study focused on the rs5707 genotype, preeclampsia is a multifactorial condition influenced by various genetic, environmental, and lifestyle factors. Comprehensive genomic studies that include a wider range of genetic markers could provide a more holistic understanding of the genetic predispositions to preeclampsia and associated outcomes.

Finally, the study did not explore the potential mechanisms by which rs5707 genotypes influence BMI and neonatal outcomes. Further research into the biological pathways involved could elucidate the underlying mechanisms and identify potential therapeutic targets for preventing and managing preeclampsia.

## 6. Conclusions

This study explored the association between rs5707 genotypes and maternal and neonatal outcomes in control and preeclampsia/eclampsia (PE/E) groups. Significant genetic predispositions linked to the rs5707 genotype were found, influencing pre-pregnancy BMI and neonatal outcomes, such as Apgar scores, birth weights, and gestational ages. The AA genotype, associated with a reduced risk of preeclampsia, was more frequent in the control group with lower BMI values, while the AC genotype was linked to an increased BMI and higher preeclampsia risk. Although the small sample size limited the statistical significance, these findings underscore the potential of genetic profiling in identifying at-risk patients and highlight the importance of considering genetic factors in managing PE/E.

## Figures and Tables

**Table 1:** Inclusion and exclusion criteria.

**Control Group**	
**Inclusion Criteria**	**Exclusion Criteria**
Pregnant women aged between 18 and 40 years old Women admitted to the Clinical Departments of Obstetrics and Gynecology of the “Pius Brinzeu” Emergency Clinical Hospital Timisoara for delivery Participants who have provided informed consent to take part in the study Participants who can understand and communicate in Romanian Participants who have attended at least two prenatal care visits during their pregnancy Newborns born at term (between 37 and 42 weeks of gestation) without any immediate life-threatening conditions Complete medical records available for both the mother and the newborn throughout the study period Women maintaining normal blood pressure levels (systolic < 140 mmHg and diastolic < 90 mmHg) throughout the pregnancy Patients having a smooth, uncomplicated pregnancy without the need for hospitalization due to pregnancy-related complications Pregnant women with no history or diagnosis of preeclampsia (PE) or eclampsia (E) during the current pregnancy	Multiple pregnancies Additional obstetrical complications, including placenta previa, abruption placentae, or placental insufficiency History of chronic or gestational hypertension recorded in the patient’s medical history Pre-existing or gestational diabetes mellitus diagnosed during pregnancy Renal conditions, such as glomerulonephritis or diabetic nephropathy, and cardiovascular conditions, such as congestive heart failure or coronary artery disease, identified in the patient’s medical history Severe congenital anomalies detected in the fetus during prenatal evaluations, including congenital heart defects or central nervous system malformations Use of teratogenic medications or known toxic substances harmful to fetal development, as well as exposure to teratogenic substances during pregnancy Alcohol consumption and active smoking during pregnancy, documented in the patient’s behavioral history Presence of other severe medical conditions that may affect the course of pregnancy and fetal health, such as autoimmune diseases or active viral infections, such as HIV or hepatitis B or C Participant’s refusal to follow the research protocol or to provide informed consent adequately for participation in the study
**PE/E Group**	
**Inclusion Criteria**	**Exclusion Criteria**
Pregnant women aged between 18 and 40 years old Pregnant women who were between 20 and 40 weeks of gestation at the time of being diagnosed with PE/E Women admitted to the Clinical Departments of Obstetrics and Gynecology of the “Pius Brinzeu” Emergency Clinical Hospital Timisoara for delivery Participants who have provided informed consent to take part in the study Participants who can understand and communicate in Romanian Participants who have attended at least two prenatal care visits during their pregnancy Newborns born at term (between 37 and 42 weeks of gestation) without any immediate life-threatening conditions Complete medical records available for both the mother and the newborn throughout the study period	Multiple pregnancies Additional obstetrical complications, including placenta previa, abruption placentae, or placental insufficiency History of chronic or gestational hypertension recorded in the patient’s medical history Pre-existing or gestational diabetes mellitus diagnosed during pregnancy Renal conditions, such as glomerulonephritis or diabetic nephropathy, and cardiovascular conditions, such as congestive heart failure or coronary artery disease, identified in the patient’s medical history Severe congenital anomalies detected in the fetus during prenatal evaluations, including congenital heart defects or central nervous system malformations Use of teratogenic medications or known toxic substances harmful to fetal development, as well as exposure to teratogenic substances during pregnancy Alcohol consumption and active smoking during pregnancy, documented in the patient’s behavioral history Presence of other severe medical conditions that may affect the course of pregnancy and fetal health, such as autoimmune diseases or active viral infections, such as HIV or hepatitis B or C Participant’s refusal to follow the research protocol or to provide informed consent adequately for participation in the study

**Table 2:** Comparative analysis of demographic and health variables between control and PE/E groups.

Variables	Control Group (N = 254) Mean ± SD*/n (%)	PE/E Group (N = 146) Mean ± SD/n (%)	*p*-Value
Maternal age (years)	27.3 ± 5.8	29.4 ± 6.3	<0.0001 *
Systolic BP (mm Hg)	119.4 ± 10.8	159.4 ± 13.8	<0.0001 *
Diastolic BP (mm Hg)	78.4 ± 12.2	109.3 ± 13.1	<0.0001 *
Education:			
- No formal education	39 (15.35%)	16 (10.95%)	0.219
- High School	62 (24.04%)	41 (28.08%)	0.372
- Bachelor’s/College Degree	146 (57.48%)	82 (56.16%)	0.797
- Post-graduate Studies	7 (2.75%)	7 (4.79%)	0.285
Marital Status:			
- Married	168 (66.14%)	84 (57.53%)	0.086
- Engaged	69 (27.16%)	51 (34.93%)	0.103
- Single	17 (6.69%)	11 (7.53%)	0.751
Occupation:			
- Unemployed	111 (43.70%)	51 (34.39%)	0.068
- Skilled worker	91 (35.82%)	54 (36.98%)	0.816
- Professional	52 (20.47%)	41 (28.08%)	0.083
Parity			
- Primipara	108 (42.51%)	71 (48.63%)	0.236
- Multipara	146 (57.48%)	75 (51.36%)	0.236
Before pregnancy BMI * (kg/m^2^)			
- Underweight (BMI < 18.5)	25 (9.84%)	5 (3.42%)	<0.0001 *
- Normal weight (BMI 18.5–24.9)	174 (68.50%)	38 (26.03%)	<0.0001 *
- Overweight (BMI 25–29.9)	35 (13.78%)	50 (34.25%)	<0.0001 *
- Obese (BMI ≥ 30)	20 (7.87%)	53 (36.30%)	<0.0001 *
Stress during pregnancy			
- Low (PSS score 0–13)	101 (39.37%)	36 (24.65%)	0.005 *
- Medium (PSS score 14–26)	76 (29.92%)	48 (38.37%)	0.084
- High (PSS score 27–40)	77 (29.96%)	62 (42.46%)	0.011 *

* Significant statistical differences, SD = standard deviation, BMI = body mass index.

**Table 3:** Association of rs5707 genotypes with preeclampsia/eclampsia in maternal and fetal groups.

Characteristics		rs5707		
		AA n (%)	AC n (%)	CC n (%)
Maternal	Control group (N = 254)	136 (53.54%)	108 (42.51%)	10 (3.93%)
	PE/E Group (N = 146)	103 (70.54%)	36 (24.65%)	7 (4.79%)
	OR (95% CI)	0.4812 (0.3121 to 0.7418)	2.2603 (1.4396 to 3.5488)	0.8138 (0.303 to 2.186)
	*p*-Value	0.0009	0.0004	0.682
	Z Statistic	3.312	3.543	0.409
Fetal	Control group (N = 254)	126 (49.60%)	107 (42.12%)	21 (8.26%)
	PE/E Group (N = 146)	98 (67.12%)	33 (22.60%)	15 (10.34%)
	OR (95% CI)	0.4821 (0.3155 to 0.7367)	2.4925 (1.572 to 3.9519)	0.7871 (0.3923 to 1.5792)
	*p*-Value	0.0007	0.0001	0.5005
	Z Statistic	3.373	3.884	0.674

**Table 4:** Association of rs5707 genotypes with maternal pre-pregnancy body mass index (BMI).

rs5707 Genotype	Control Group		PE/E Group		OR (95% CI)	*p*-Value
	BMI < 24 kg/m^2^ (N = 199) n (%)	BMI ≥ 24 kg/m^2^ (N = 55) n (%)	BMI < 24 kg/m^2^ (N = 43) n (%)	BMI ≥ 24 kg/m^2^ n (%)		
AA	120 (60.30%)	16 (29.09%)	20 (46.51%)	50 (48.54%)	18.75 (8.9858 to 39.1241)	<0.0001
AC	65 (32.66%)	32 (58.18%)	18 (41.86%)	45 (43.69%)	5.0781 (2.5437 to 10.1379)	<0.0001
CC	14 (7.04%)	7 (12.73%)	5 (11.63%)	8 (7.77%)	3.2000 (0.7587 to 13.4974)	0.113

**Table 5:** Association of maternal rs5707 genotypes with Apgar score, birth weight, and gestational age at birth in control and PE/E groups.

rs5707 Genotype	Apgar Score (Mean ± SD)		Birth Weight (Mean ± SD)		Gestational Weeks	
	Control Group	PE/E Group	Control Group	PE/E Group	Control Group	PE/E Group
AA	8.5 ± 1.1	7.8 ± 1.2	3211 ± 403 g	2932 ± 457 g	39.2 ± 1.1	37.5 ± 1.8
*p*-Value	<0.0001		<0.0001		<0.0001	
AC	8.3 ± 1.2	7.6 ± 1.4	3099 ± 391 g	2853 ± 503 g	39.0 ± 1.2	37.2 ± 2.0
*p*-Value	0.005		0.003		<0.0001	
CC	8.2 ± 1.1	7.4 ± 1.5	3050 ± 429 g	2803 ± 396 g	38.8 ± 1.3	37.0 ± 2.1
*p*-Value	0.0732		0.0878		0.0032	

## Data Availability

The raw data supporting the conclusions of this article will be made available by the authors on request.
